# Use of a simulated patient case and structured debrief to explore trainee responses to a “non-compliant patient”

**DOI:** 10.1186/s12909-022-03894-7

**Published:** 2022-12-06

**Authors:** Waseem Sous, Kay Frank, Peter Cronkright, Lauren J. Germain

**Affiliations:** 1 Department of Internal Medicine, SUNY Upstate Medical University, Syracuse, NY USA; 2 Department of Hospital Medicine, University of California, San Francisco, San Francisco, CA USA; 3 Present Address: HEAL Initiative, University of California, San Francisco, San Francisco CA, USA; 4 Department of Public Health and Preventive Medicine, SUNY Upstate Medical University, Syracuse, NY USA

**Keywords:** Non-compliant, Dehumanization, Doctor-patient relationship, Refugee, Simulated patient

## Abstract

**Background:**

Labeling a patient “non-compliant” is a form of dehumanization that can deprive the patient of positive human qualities and/or agency in the mind of a physician. The term “non-compliant” is frequently used in medical record documentation and has been shown to compromise care, particularly for marginalized communities. There is limited literature on the impact of the label on medical trainees. We aimed to explore how internal medicine residents and fellows (trainees) perceive the term “non-compliant patient” and its impact on their practice after interacting with a simulated refugee patient who has not followed a physician’s recommendations.

**Methods:**

Kolb’s experiential learning cycle guided the design of the educational session which was part of a required communication skills curriculum for trainees. A scenario was created to simulate a refugee patient who had not adhered to their treatment plan and could potentially be labeled as “non-compliant.” Trainees participated in the 3-h session consisting of a remote simulated patient encounter immediately followed by a virtual structured debrief session that was recorded and transcribed. Thematic analysis of debrief transcripts was conducted starting with the use of provisional codes from the literature on the doctor-patient relationship and de/humanization.

**Results:**

In group debrief sessions, trainees reflected upon the standardized patient case and chose to also discuss similar cases they had experienced in clinical practice. Trainees indicated that the term “non-compliant patient” served as a biasing function and described how this bias negatively impacted the doctor-patient relationship. Trainees described how marginalized communities might be more susceptible to the negative connotation associated with the term “non-compliant patient.” For some trainees, the term triggered further investigation of underlying barriers to care and exploration of the social determinants of health.

**Conclusions:**

The use of the phrase “non-compliant patient,” though common in medical practice, may lead to patient dehumanization among trainees. A simulated refugee patient encounter followed by a facilitated group debrief allowed participants to verbalize and reflect on the meaning and possible impact of the label.

**Supplementary Information:**

The online version contains supplementary material available at 10.1186/s12909-022-03894-7.

## Background

Labeling a patient “non-compliant” is a form of dehumanization which can deprive the patient of positive human qualities and/or agency in the mind of a physician [1]. Amen *et al. *[2] highlight how medical education can unintentionally lead to patient dehumanization when terms such as “frequent flyer” and “non-compliant” are used as patient descriptors. Though use of the term “non-compliant patient” has been found to have negative consequences on patient care [3, 4], particularly for marginalized communities [5], the descriptor is frequently used in medical record documentation [5]. Since refugees often face a unique set of barriers to accessing care, including language, education, lack of insurance, and transportation [6], they may be especially vulnerable to such patient descriptors that negatively impact their care. 

The physician authors (PC, WS) noted that the term “non-compliant patient” was a label used frequently during resident physicians’ case presentations of refugee patients and wondered about the potential for the vernacular to lead to patient dehumanization and affect the doctor-patient relationship. The use of standardized patient (SP) encounters can provide opportunities to explore cultural diversity in healthcare delivery [7–13] and offer controlled patient experiences with space for clinician reflection and feedback in a supportive environment [14–16]. Reflection gives physicians chances to enhance their clinical skills, practice cultural humility, recognize structural vulnerability [17] and social determinants of health [18], and decrease their own anxiety [19]. Thus, a simulated refugee patient case was developed by the physician authors and was incorporated into a communication skills curriculum at the State University of New York (SUNY) Upstate Medical University. This SP curriculum, known as Learning to TALK (Treat All Like Kin), was developed to enhance medical trainees' communication skills, professionalism, and clinical competencies.

Given the limited literature on the impact of the “non-compliant” label on medical trainees, we aimed to explore the questions: 1) how do internal medicine residents and fellows (trainees) perceive the term “non-compliant patient?”; and 2) in what ways does the term impact the doctor-patient relationship after interacting with a simulated refugee patient who has not followed a physician’s recommendation?

Todres, Galvin, and Holloway [20] offer a conceptual framework describing eight dimensions of humanization and suggest that dehumanization can occur when any one of the humanizing forms is obscured to a significant degree. Forms of dehumanization include objectification, passivity, homogenization, and reductionist body; each is defined including associated behaviors and their impact on patient care [20].

Szasz and Hollender’s schema categorizes doctor-patient relationships into three forms: activity-passivity, guidance-cooperation, and mutual participation [21]. In the activity-passivity model, the physician “does something to the patient” and resembles the parent-infant relationship. The guidance-cooperation model has the qualities of a parent-adolescent relationship and in it, the physician “tells the patient what to do.” Finally, in the mutual participation model, the physician and patient work in partnership as two adults.

## Methods

### Context

Medical trainees in the Department of Medicine at the SUNY Upstate Medical University participate in a required simulated patient (SP) curriculum aimed at improving communication skills. Kolb’s experiential learning cycle [22] guided the design of the teaching sessions, which begins with the concrete experience of an SP encounter that is both recorded and observed by faculty. Following each encounter, trainees participate in a group debrief where they reflect with the guidance of a trained facilitator. After completion of the group debriefs, trainees proceed to watch the SP encounter video.

### SP encounter

Given the COVID-19 pandemic, the SP encounters and debriefs for this study were performed virtually. Between December 2020 and January 2021, trainees participated in a case of a refugee patient with hypertension and diabetes. Each SP encounter lasted for approximately 15 min. The script described an English-speaking refugee from Syria with uncontrolled hypertension and diabetes (Appendix A). The setting involved an acute ambulatory care visit, occurring 3 days after the patient was assessed and managed in the emergency room (ER) for headache, hypertensive urgency, and uncontrolled diabetes. SPs received training and observation by the SP program director to ensure consistent patient experience (Appendix B). The case simulates the real-world context including time pressure for the provider and includes references to various social determinants that influence the patient’s ability to follow the care plan. Trainees were expected to: 1) address the patient’s uncontrolled hypertension and diabetes; 2) determine the appropriateness of temporary disability; 3) consider recommended health maintenance screenings and/or immunizations.

### Data collection

Facilitated discussions among trainees during structured debrief sessions were used to collect data due to the exploratory nature of the study and the integral role of group interaction in the medical training experience. After each SP encounter, trainees participated in a structured virtual group debrief session led by a trained facilitator. The debrief protocol (Appendix C) was developed to prompt trainees to reflect on their encounter experiences, specifically as they relate to the term “non-compliant patient” and their perceptions of the associated doctor-patient relationship. Debriefs were recorded, transcribed using Otter.ai [23], and checked for accuracy by an author (WS). Transcriptions showed that group debriefs followed the script provided to facilitators to ensure a structured and standardized discussion. All trainees completing the SP experience were invited to participate and comments by trainees who did not consent to the project were removed from the transcript by WS.

### Analysis

A team of four, two physicians (WS, PC) and two social scientists (LG, KF), developed the provisional code list from Todres *et al*.’s [20] work on de/humanization and Szasz and Hollendar’s [21] work on the doctor-patient relationship. NVivo Qualitative Data Analysis Software (QDAS) [24] was used for thematic coding. Coding was conducted simultaneously by all four authors who discussed all coding until 100% agreement was reached. Results were shared with a physician who is active in resident training and specializes in the care of refugee patients to work toward credibility [25]. The project was granted exemption by the SUNY Upstate’s Institutional Review Board.

## Results

In total, five virtual post-session facilitated group reflections were held. Forty-two internal medicine categorical interns and second- and third-year residents (39% of the program) and 8 fellows (15% of all fellows) participated in the SP encounters. Participants represented 56% of categorical interns (20/36), 36% of second-year residents (13/36), and 25% of third-year residents (9/36). The fellows represented 6 internal medicine subspecialities: cardiology, gastroenterology, endocrinology, geriatrics, infectious diseases, and rheumatology. 46 (92%) trainees consented to have their debrief participation recorded.

### Negative connotation and dehumanization

Within debrief sessions, the phrase “non-compliant patient” was frequently described as carrying a negative connotation and was connected to patient dehumanization. For example, one trainee said, “overall, when you read this non-compliance thing and how the guy skipped an appointment and is looking for the work letter, you can't help but go into the encounter thinking it will be a more resistant or a jerk back to you…” A trainee commented, “It doesn't sound nice to say this, but at the same time if you feel that your patient doesn't care (because of the non-compliance) it makes you feel like, ‘should I care if they don't care?' Not that that's the case but it really makes you think about that. Why am I doing what I'm doing if the patient doesn't even want to get what they need?” Another stated, "When I see the word non-compliant in the chart, it automatically changes my prognosis that I have set for the patient is what I would say… and maybe on a subconscious level, I won't be trying as hard for this particular patient."

A few trainees humanized and connected to patients by considering their feelings about being labeled. One trainee commented about how they would feel if they had been labeled “non-compliant” in their chart and suggested asking patients for their feelings:

- I think it's probably going to be interesting to see what happens when patients see that in their medical chart when they are able to review their notes soon. I think if I was a patient and read “non-compliant” in my chart, I would probably be offended. I mean, I definitely would be. I think there are probably better terms that we could use. We spend a lot of time drilling in what type of heart failure we have when we put that in a chart. We want to be as specific as possible. I don't know, it's just my personal opinion. Perhaps we should consider a different terminology. We can poll patients and see how they feel about the term.

Another reflected on how it might feel to be labeled and what the term tells the patient about their role in the doctor-patient relationship:

- ...if I was a patient and someone said that I was non-compliant. I mean, if you think about it, to have to comply with somebody is like you're being dictated to do something. So from the patient's perspective, it shouldn't be a dictation from the doctor. You must do this. I think it should be a conversation. So, I think that would kind of give them the impression that I don't see myself as someone that's working with them for their health, more so as someone that's dictating what they're going to do…

### Impact on the doctor-patient relationship

Trainees described the dynamics of the relationship between themselves and the patient labeled as “non-compliant” in three ways which align with a previously published schema (Table 1) [21]. The activity-passivity model was described very rarely in the debrief sessions. When it was described, the comment was to dehumanize a patient in a theoretical case of activity-passivity:

- I do not think it was the case with this patient, but I think often it happens more when it is a translator patient. When you try to explain what you want to prescribe or the changes you want to make, I feel like sometimes the patients just tend to nod and say okay, but you have no idea if they are ever going to follow those recommendations. Sometimes you think you are just maybe talking to a wall because certain cultures do not want to disagree with what the doctor...

More commonly, trainees took the stance of “guidance-cooperation.” These trainees described the patient in dehumanizing and/or humanizing terms with similar frequency (Table 1), sometimes speaking about the SP case and other times reflecting on their experiences overall. One trainee spoke in generalities about a guidance-cooperation stance that humanized patients, “I think if patients are motivated, you have to figure out or come to a plan with them and realize that it’s not that they don’t want to be treated, it’s maybe they don’t know how to approach it. Then you can deal with the situation.” Another trainee spoke in generalities about a guidance-cooperation stance that dehumanized patients, “…There are some patients that will be non-compliant no matter what you do for them. But you should always start with the assumption that there is a possibility of “converting” this patient to become compliant again…”

Those who adopted the mutual participation relationship model did not use dehumanizing terms and often investigated the underlying social determinants of health [18] (Table 1). For example:

- You're trying to optimize their health and there are multiple factors holding them back from being able to do that. So for [SP name] it could have been anything from his culture to lack of literacy or education or social support… He mentioned to me, he wasn't taking one of his blood pressure medicines because it was interfering with him at work. It was making him use the restroom way too frequently for his comfort. So we always want to be able to develop a plan that works for us but also one that works for the patient.

### De/humanization and the doctor-patient relationship

Medical trainees noted how the term “non-compliant patient” can serve as a biasing function that risks patient dehumanization and impacts the doctor-patient relationship, as noted in the following four examples: 1) “I think it creates a barrier for a good doctor-patient relationship. It's very easy to just read non-compliant and say, ‘Oh, you know this is going to be a difficult encounter. I'm going to have a hard time with him,’ so you'll just be predisposed to that from the start;” 2) “I just feel that non-compliance is such a negative word. I don't think that it should be used as much as we use it because it creates a very negative impression about a patient. It blames the patient… I think non-compliant is a very strong word to be used and I guess we should use it more carefully when describing anybody;” 3) “I think we're also very vulnerable to being biased. You know when someone says this is a non-compliant patient coming here again with another COPD exacerbation, I think it is human to say: What am I going to do differently? I think that does play a big role in how you approach a patient. I think if someone just comes and tells you this is a non-compliant patient, you no longer see what he's up to;” 4) “Big pet peeve because negative connotation comes to it, and oftentimes we just say it's just a difficult patient rather than trying to consider what the reason is. Oftentimes, we just say take this medication and we don't consider how it might impact someone's personal life. I think that's the underlying issue. So, labeling patients can become problematic, and we pass it along from provider to provider. We're in a rush walking into a room, and we say: ‘It's another non-compliant patient.'”

### Perception changes

Many trainees recognized the potential negative consequences of using the term after participating in the SP encounter, despite previously using the term as a “flag” to “dig” into a patient's history:

- I have used that frequently and I've heard other people use it as well. To me it kind of stands out as a red flag. Sometimes you do have patients that are frustrating and don't seem to care much about their health, but oftentimes I use it as a red flag to alert myself that the patient may have barriers to health care, and it helps me dig deeper. When I was speaking with the patient, I used the word and discussed compliance with him. So I could see kind of how it could come off negatively to a patient or someone, and that maybe avoiding that term and finding a different word for it would be a bit more appropriate.

Some trainees noted a change in their impression of the SP encounter, indicating an initial negative emotion that transitioned to a more positive one after collaborating with the SP:

- I felt pretty good after figuring out the side effects and we came up with a better plan for how we're going to re-adjust. At the end, we had much better rapport... In the beginning, I was borderline judging a little bit and thinking he was going to be non-compliant, but in the end I felt a lot better about it.

Other trainees described the impact of the SP encounter on their future clinical behavior as noted in the following example:

- The term non-compliant has a bad connotation to it and I felt that that did play a part in my encounter with him... I realized that instead of delving more into what exactly was the reason behind his non-compliance, I spent a few extra minutes going over the risks and consequences of him being non-compliant... I think that is definitely something that I'll keep in mind going forward.

### Impact on marginalized communities

A few trainees described how marginalized communities might be more susceptible to the negative connotation associated with the term “non-compliant patient.” For example:

- A lot of times, we assume that especially for patients who are immigrants or those with language barriers. We kind of jump into conclusions very prematurely. And then if you just listen to them patiently and then you can get to the bottom of “why was he worried about losing his job?” or “why has he not been taking the medicine properly?”

In the following cases, trainees seemed to actively “dig” into the patient’s history and possible underlying causes for not following a physician’s recommendation, despite using the label:

- The word non-compliant doesn't always have to be a bad term. When you use it to say, all right, they are non-compliant, let's figure out why. And I think making sure that we use that term to say we have to dig deeper and use the resources that we have… Some of my patients in clinic have had problems where they don't know English, they don't know which medications to take, what the bottles look like, and what they're supposed to take and when. And when I've gotten them the dispill packs, I've seen significant success. This doesn't work for every patient, it doesn't fix every patient's problem, but trying to figure out why is really important.

## Discussion

Negative patient descriptors such as “non-compliant patient” are documented in medical records and have been shown to compromise care, particularly for marginalized communities that face significant barriers to healthcare [5]. Additionally, use of the term “non-compliant patient” has been cited as a negative connotation with resultant negative consequences to patient care [3, 4]. Thus, labeling a patient “non-compliant” is a form of dehumanization which can deprive the patient of positive human qualities and/or agency in the mind of a physician [1]. Given the limited literature on the impact of this label on medical trainees, this study utilized a refugee simulated-patient encounter, followed by a facilitated group debrief session, prompting trainees to reflect on their encounter experiences. The educational session was analyzed to determine whether a physician’s verbalization of the “non-compliant patient” or the perceived action of “non-compliance” might lead to patient dehumanization and negatively impact the doctor-patient relationship.

Trainees acknowledged that the term “non-compliant patient” was used commonly in clinical practice to describe a patient and is often perceived as a biasing function that risks dehumanization and impacts negatively on patient care. Trainees described how marginalized communities, particularly refugees and immigrants, might be more susceptible to the negative connotation associated with the term “non-compliant patient.”

Trainees described the encounter in different ways, but most commonly they described the doctor-patient relationship as one of mutual participation or guidance-cooperation. The activity-passivity model was described very rarely in the debrief sessions; thus it is difficult to draw an association between the use of dehumanizing terms by trainees who took an activity-passivity stance. Trainees who adopted the mutual participation care model did not use dehumanizing terms and highlighted how the label can impact patient care. Since the mutual participation care model emphasizes a partnership between the physician and patient [21], decisions are likely to be made by patients based on their preferences and social circumstances. Thus, participation in such a care model can minimize, and possibly eliminate, the use of dehumanizing terms. Trainees who adopted the guidance-cooperation model used dehumanizing and humanization terms with similar frequency, which illustrates the possibility in which the term “non-compliant patient” can trigger the physician to question why and explore the underlying reasons behind not following a physician’s recommendation. In the guidance-cooperation model, the patient is a semi-active participant and tends to follow a physician's recommendation regarding the treatment plan [21]. Since this model has the qualities of a parent-adolescent relationship [21], there is a power differential dynamic that can potentially result in dehumanizing terms. It is interesting to note that some of the trainees who utilized the term as a “flag” to “dig” into a patient’s history and underlying barriers to care, also highlighted the potential negative consequences associated with it. Thus, faculty mentors are encouraged to use the term as a signal to explore further: (1) the contextual factors that may affect medication adherence, which trainees often cite as being outside the provider’s or patient’s control; (2) the risk and impact of the term on the doctor-patient relationship; and (3) the risk for patient dehumanization when using the term.

Some trainees noted an impression transition during the SP encounter and others demonstrated self-reflective behavior for potential change in future clinical encounters. This educational study design not only aligns with Kolb’s experiential learning cycle [22] as it highlights the potential for change in future clinical behavior through active experimentation, it also illustrates the importance and impact of the group debrief as a safe environment for discussion and reflection [16]. Moreover, it exposes the potential impact of using the term while describing refugee patients who face significant barriers to care [6], and allows trainees to reflect on the underlying social determinants of health that can impact care [18].

Future studies should explore the potential impact of time-constraints on learners' tendency to use patient labels, such as “non-compliant patient”, which may lead to biased rapid clinical decision-making. Additionally, future studies should focus on options for systems-based change that support the provider and advocate for the patient, capture the impact of such an educational experience on medical trainees' likelihood of using the term and future clinical behavior, and explore the possible role of the term that may allow trainees to elucidate the underlying barriers to care [6] and social determinants of health [18].

### Limitations

Although this SP encounter was part of a required communication skills curriculum in the Department of Medicine, the cases are typically rotated and this case was held for a total of five sessions. As such, 31% (50/162) of the possible interns, residents, and fellows were eligible to participate. Additionally, sampling was based on trainee clinic schedules during December and January when interns are less likely to be on vacation than their counterparts in the second year and above, so they were slightly overrepresented in the sample.

The data were coded in transcripts rather than on video, which limited understanding of non-verbal communication cues. Additionally, comments by trainees who did not consent to the project were removed from the transcripts leaving some gaps in the conversations. Another limitation in this study is priming in the use of the protocol. Debrief sessions occurred immediately after the SP encounter and in the groups, some trainees immediately noted that the use of the term “non-compliant patient” may impact the doctor-patient relationship.

## Conclusions

This simulated refugee patient encounter followed by a facilitated peer-group debrief allowed participants to verbalize and reflect on the meaning and possible impact of using the label. Trainees acknowledged that the term “non-compliant patient” was used commonly in clinical practice to describe a patient and is often perceived as a biasing function that risks dehumanization and impacts negatively on patient care. Trainees who adopted the mutual participation care model did not use dehumanizing terms. Trainees who adopted the guidance-cooperation model used dehumanization and humanization terms with similar frequency, which demonstrates the possibility that the term “non-compliant patient” might trigger further investigation of underlying reasons for not following a physician’s recommendation. Finally, faculty mentors can play an important role in the recognition of the term when used by trainees, which can help promote a change in future clinical behavior through active experimentation, aligning with the final step in Kolb’s experiential learning cycle [22].

**Table 1:** Models of doctor-patient relationship [21] and its connection to dehumanization and humanization [20]

**Model**	**Connection to dehumanization**	**Connection to humanization**
Activity-Passivity	Cited rarely	Never
Guidance-Cooperation	Cited in 4/5 with similar frequency to connection to humanization. “I think a lot of the times when we go into these rooms, we anticipate the patient not having any kind of motivation to want to change or be interested in listening about what to do. They would just give you a head nod and move…” “...we can help with all services to make sure that he gets the necessary treatment needed and after all those measures, if the patient is just not willing to take the medication, then I would label him as non-compliant or whatever and would assume that it is a very difficult interaction to be had…”	Cited in 4/5 with similar frequency to connection to dehumanization. “I was inquisitive. I wanted to see what problems he was having that were causing him to have these work difficulties, and he quickly explained it…I had a feeling he was going to meet the definition of what we call non-compliant. My goal was to see how we can optimize his medication regimen.” “It also depends on the mindset of the provider…some people when they see non-compliant, they might say: why am I seeing this person. Other people might have the mindset of: I wonder why they are not taking their medications…You the opportunity to try to establish your own connection with the patient and figure out why he does not take the medication…”
Mutual Participation	Never	Common - cited in 5/5 debriefs. “I think that sometimes when we are told about a patient, and non-compliance comes up, I think some people will see it as an easy way out…I think it is harder to figure out what the problem is: why are people not compliant? Can they afford the medication? Are they having a side effect they cannot tolerate? Are they having difficulty going to the pharmacy? Ultimately, that leads to better patient outcomes and rapport with patient.” “...It wasn't that he didn't care about his health…or put an effort. It was more so he had all these barriers. And it was our job as a team to figure out a way that we could make a plan that would work for both us as a provider and him as a patient…”

## Supplementary Information

**Additional file 1**.

## Data Availability

The datasets generated and/or analyzed during the current study are not publicly available to ensure that trainees’ privacy is not compromised but are available from the corresponding author on reasonable request.

## Abbreviations

ER: Emergency Room
SP: Simulated Patient
QDAS: Qualitative Data Analysis Software
SUNY: State University of New York
