# Correction: Use of a simulated patient case and structured debrief to explore trainee responses to a “non-compliant patient”

**DOI:** 10.1186/s12909-022-03962-y

**Published:** 2022-12-19

**Authors:** Waseem Sous, Kay Frank, Peter Cronkright, Lauren J. Germain

**Affiliations:** 1 Department of Internal Medicine, SUNY Upstate Medical University, Syracuse, NY USA; 2 Department of Hospital Medicine, University of California, San Francisco, San Francisco, CA USA; 3 Present Address: HEAL Initiative, University of California, San Francisco, San Francisco, CA USA; 4 Department of Public Health and Preventive Medicine, SUNY Upstate Medical University, Syracuse, NY USA

**Correction: BMC Med Educ 22, 842 (2022)**

**https://doi.org/10.1186/s12909-022-03894-7**

Following publication of the original article [1], we have been informed that author Kay Frank’s affiliation has not been added.

The author affiliation has been updated above and the original article has been corrected.
