# Supplementary material for: Use of a simulated patient case and structured debrief to explore trainee responses to a “non-compliant patient”
Source: BMC Med Educ. 2022 Dec 6;22:842. doi: 10.1186/s12909-022-03894-7 (PMC9727840; doi:10.1186/s12909-022-03894-7)
Supplement: Supplementary file 1 — Additional file 1. [file 12909_2022_3894_MOESM1_ESM.docx]

**APPENDIX**

**A**

**Script provided to trainees prior to the SP encounter:**

Patient Problem:      Hypertension and Diabetes

You are at Upstate Health Care Center (UHCC) today seeing a combination of in-person and telemedicine visits.  It’s 10:50AM and you just finished with your last patient of the morning.  You’ve just now been alerted that Amal Mohammed is a 52 y.o. patient seen at UHCC in the past (not by you) who has been added to your schedule as a telemedicine urgent add-on for 11AM.  The patient is an English-speaking refugee from Syria with a history of difficult-to-control hypertension on Losartan and Hydrochlorothiazide as well as diabetes (diagnosed upon resettling in the United States about a year ago) and takes Glargine and Metformin.  Records indicate the patient has a home blood pressure (BP) machine.

Review of EPIC Electronic Health Record and Regional Health Information Organization (RHIO) reveals the patient was discharged 3 days ago after an ER visit for headache, hypertensive urgency, and uncontrolled diabetes.  BP on arrival to ER was 205/114mmHg.  Physical exam was normal including cardiac and neurologic exams.  Glucose 288mg/dL; Potassium 3.2meq/L; Creatinine 1.4mg/dL; EKG with LVH unchanged from prior.  BP responded to oral Clonidine.  BP at discharge was 148/100.  The prior oral home medications were resumed plus the ER doctor prescribed the following additions: Amlodipine 5 mg daily, Potassium Chloride (KCl) 10 mEq daily, and Glargine Insulin was increased from 20 to 26 units nightly.  The patient was a no-show for the scheduled in-person follow-up appointment yesterday, but the patient’s son called today demanding that the patient be seen for employment reasons.  The nurse’s note indicates the patient offers no medical complaints, but needs a note for work and couldn’t find transportation to come into the office.  It’s now 11:25AM and registration and nursing have completed their “rooming” requirements.  Patient-Reported Vitals:  BP-180/110mmHg P-90b/m.

Epic Medication List:  Losartan 100 mg daily, Hydrochlorothiazide 25 mg daily, Glargine Insulin 20 Units nightly; Metformin 1000 mg twice daily;  Atorvastatin 40 mg daily.  Trazodone 100 mg nightly.  You called the pharmacy and confirmed that the patient picked up their medications since discharge from the ER.

Epic chart review notes an A1c in 4/03/2020 was 7.0 %.  Social History:  Smokes 3 cigarettes daily; divorced, lives alone, has 1 son (age 31); Muslim religion; denies recreational drug use; refugee from Syria.

Of note, it is now 11:30AM and you are hoping to catch lunch before boarding the shuttle to Community Campus for your afternoon subspecialty service.

In this encounter you are expected to do the following:

1. Address the patient’s uncontrolled hypertension and diabetes.
2. Determine if a letter for missing work is appropriate and when the patient may safely return to work.
3. Review required health maintenance including need for colonoscopy and vaccinations.

**APPENDIX**

**B**

**Guide used to train the SPs:**

**Amal Mohammed (SP name)**

You are an English-speaking refugee from Syria who came to the United States about one year ago.  You’ve had hard-to-control high blood pressure (hypertension) for a few years and were diagnosed with diabetes when you came to the United States.  You have a home BP machine that you don’t routinely use.  You work as a parking attendant.

You went to the Crouse ER three days ago for a headache after working a double shift.  You spent the night in the ER for very high blood pressures and high sugars (you don’t remember the exact values; you just remember being told “they were high”).  Some new medications were added and your insulin dose was adjusted.  You were discharged the following day which was a scheduled day off from work.

Following the ER visit, you obtained the new medications at the pharmacy.  That evening you took one of every medicine and injected 26 units of insulin (called Glargine) as instructed.  Tired after a night in the ER and needing to be at work before 6 am the next day, you went to bed early without eating.  On rising the following morning (yesterday), you were dizzy, weak, and felt a “fever.”  You did not go to your scheduled shift at work, and you stopped all your medications because the medications made you sick.  You felt this “fever” before when fasting and taking your medications during Ramadan such that you stopped your medications then and felt better.

Before you left the ER, they had made a follow-up in-person visit with your doctor scheduled for yesterday.  You forgot you had that appointment and did not go to it.

Someone from work called you and wanted to know why you missed a day at work.  You told them that you were sick but can work now.  You had not called in sick to work.  They said that you need a letter from your doctor for being sick from work or you could lose your job.

Your son assisted you in calling your doctor’s office and explained you need an urgent appointment, but don’t have the means to get to the office unless transportation can be arranged.  They offered you an opportunity to see a covering doctor over the internet.  You’ve never done a doctor’s visit like this before, but you do know how to use the internet and agree to the visit as you must get the note for work today or you could lose your job.

You live alone in your own apartment.  You are currently at your son’s apartment (so you don’t have access to your medications, if asked).  He helps you translate because his English is better than yours.  He is at work now and not available to speak with.  You are divorced and your ex-wife is still in Syria).

The nurse you spoke with a few minutes ago had you take your blood pressure and pulse on your home machine; your blood pressure was 180/110 and your pulse was 90.

A resident doctor connects with you via the internet.  You tell him/her that “someone from work told me you would give me a work note.”  You don’t know the official job title or phone number of the person who called you.

If the doctor asks about your blood pressure, you tell them your blood pressure was elevated because you do not take any pills on work days as they make you urinate and you cannot leave your workstation.

If the doctor asks about your diabetes or “fever”, you explain that you stopped your nightly insulin because you hadn’t eaten and you’ve done this in the past as well when you have had “fever”.  You know the insulin caused the fever because the fever symptoms resolved when the insulin was stopped.  Only if the resident asks about details of these prior “fever” occurrences do you volunteer that it happened during Ramadan.

If asked, you decline an interpreter because you did not trust the Shiite Arabic interpreter that you had in the past.  You don’t have your medicines with you, as you are at your son’s apartment.  You were previously given a pill box but had difficulty reading the bottle labels.  Your son fills the pill box for you and you try to take the medication correctly.  You speak fair English and count money but do not read English.  You were a baker in Syria, only completed grade 4, and struggled to read Arabic.

If the doctor offers advice on how to improve your blood pressure, you refuse the advice unless the doctor specifically offers advice on how to reduce the risk of having to urinate during work.

If the doctor offers advice on how to improve your blood glucose/diabetes, you tell them that the shot makes you sick.  If the doctor educates you that the “fever” was due to low blood sugar due to your altered schedule yesterday and during Ramadan, you are willing to restart the insulin shots but at a lower dose.

If the doctor offers advice on cancer screening or vaccinations, you accept their advice.

If the doctor agrees to provide you a note, you thank the doctor but explain you need the note today or you’ll lose your job.  If the doctor offers to write the note and have it available for you to pick up in the office, you say that you can do that on your way to work later today.   If the doctor offers to fax the note directly to your supervisors, share with them the fax number below.  If the doctor presses you on having to find a way in to get the note, you start to get frustrated.  If the doctor offers another option (such as calling your work directly), you thank them for being such a great doctor.

If the doctor refuses to give you a note, you resist and keep asking for a note.  If they continue to refuse to give you a note, you end the meeting saying you’ll call back and talk to a different doctor who cares about you.

Brief Information about Ramadan:^1^

Muslims around the world fast for the holy month of Ramadan.  This involves abstaining from food and drink from dawn (suhoor) to dusk (iftar), for the entire month, and is fundamental to the faith as one of the five pillars of Islam.^1^

*Exceptions (Who is exempted from fasting during Ramadan?)*^1^

Acute or chronic health conditions (where fasting may place them at risk of ill-health).

People in the course of traveling.

Pregnant women.

Nursing mothers.

**APPENDIX**

**C**

**Facilitator guide to questions used during the debrief sessions:**

1. When encountering patients that do not adhere to their medications or care plan, have you described such patients to your attending or in your encounter note as a noncompliant patient?
   1. If so, does the “noncompliant patient” description assist you in better understanding the patient’s needs and development of an effective care plan?
   2. Is describing the patient as “noncompliant” likely to elicit a physician’s trust or distrust in the patient?
   3. How might the description of the “noncompliant patient” hinder your doctor-patient relationship?
   4. How might the description of the “noncompliant patient” impact on your intent of preserving the patient’s dignity?  Hinder? Help? No impact?  Why do we care?
   5. Could the description of “noncompliant patient” alienate some physicians, eliciting potential stigma or stereotypical biases?
2. Have you found it difficult to work with patients that do not adhere to their medications and care plan?
   1. What emotions did you feel before going into the examining room to see this patient?
   2. What emotions did you feel immediately after the encounter?
   3. Have I felt this way before with other patients?
   4. Would anyone react in this way to the same patient?
3. What factors likely have an impact on this patient’s difficulties in taking the prescribed medication regimen?
   1. What structural issues^2^, either in or outside the visit, contribute to the challenges of providing care for this patient?  (literacy, language, complexities of our healthcare system, etc.?)

References:

1. Hanif S, Ali SN, Hassanein M, Khunti K, Hanif W. Managing People with Diabetes Fasting for Ramadan During the COVID-19 Pandemic: A South Asian Health Foundation Update. *Diabet Med*. 2020;37(7):1094-1102. https://doi:10.1111/dme.14312.

2. Bourgois P, Holmes SM, Sue K, Quesada J. Structural Vulnerability: Operationalizing the Concept to Address Health Disparities in Clinical Care. *Acad Med*. 2017;92(3):299-307. https://doi:10.1097/ACM.0000000000001294.
